# Influence of cardiac autonomic neuropathy on cardiac repolarisation during incremental adrenaline infusion in type 1 diabetes

**DOI:** 10.1007/s00125-020-05106-7

**Published:** 2020-02-07

**Authors:** Alan Bernjak, Elaine Chow, Emma J. Robinson, Jenny Freeman, Jefferson L. B. Marques, Ian A. Macdonald, Paul J. Sheridan, Simon R. Heller

**Affiliations:** 1 Department of Oncology & Metabolism, University of Sheffield, Medical School, Beech Hill Road, Sheffield, UK; 2 INSIGNEO Institute for in silico Medicine, University of Sheffield, Sheffield, UK; 3 Department of Infection, Immunity and Cardiovascular Disease, University of Sheffield, Sheffield, UK; 4 Sheffield Teaching Hospitals NHS Foundation Trust, Sheffield, UK; 5 Present Address: Department of Medicine and Therapeutics, the Chinese University of Hong Kong, Hong Kong, China; 6 Leeds Institute of Life Sciences, University of Leeds, Leeds, UK; 7 Present Address: Institute of Biomedical Engineering, Department of Electrical and Electronic Engineering, Federal University of Santa Catarina, Florianópolis, SC Brazil; 8 School of Life Sciences, Queen’s Medical Centre, University of Nottingham, Nottingham, UK

**Keywords:** Adrenaline infusion, Cardiac autonomic neuropathy, Cardiac repolarisation, Type 1 diabetes

## Abstract

**Aims/hypothesis:**

We examined the effect of a standardised sympathetic stimulus, incremental adrenaline (epinephrine) infusion on cardiac repolarisation in individuals with type 1 diabetes with normal autonomic function, subclinical autonomic neuropathy and established autonomic neuropathy.

**Methods:**

Ten individuals with normal autonomic function and baroreceptor sensitivity tests (NAF), seven with subclinical autonomic neuropathy (SAN; normal standard autonomic function tests and abnormal baroreceptor sensitivity tests); and five with established cardiac autonomic neuropathy (CAN; abnormal standard autonomic function and baroreceptor tests) underwent an incremental adrenaline infusion. Saline (0.9% NaCl) was infused for the first hour followed by 0.01 μg kg^−1^ min^−1^ and 0.03 μg kg^−1^ min^−1^ adrenaline for the second and third hours, respectively, and 0.06 μg kg^−1^ min^−1^ for the final 30 min. High resolution ECG monitoring for QT_c_ duration, ventricular repolarisation parameters (T wave amplitude, T wave area symmetry ratio) and blood sampling for potassium and catecholamines was performed every 30 min.

**Results:**

Baseline heart rate was 68 (95% CI 60, 76) bpm for the NAF group, 73 (59, 87) bpm for the SAN group and 84 (78, 91) bpm for the CAN group. During adrenaline infusion the heart rate increased differently across the groups (*p* = 0.01). The maximum increase from baseline (95% CI) in the CAN group was 22 (13, 32) bpm compared with 11 (7, 15) bpm in the NAF and 10 (3, 18) bpm in the SAN groups. Baseline QT_c_ was 382 (95% CI 374, 390) ms in the NAF, 378 (363, 393) ms in the SAN and 392 (367, 417) ms in the CAN groups (*p* = 0.31). QT_c_ in all groups lengthened comparably with adrenaline infusion. The longest QT_c_ was 444 (422, 463) ms (NAF), 422 (402, 437) ms (SAN) and 470 (402, 519) ms (CAN) (*p* = 0.09). T wave amplitude and T wave symmetry ratio decreased and the maximum decrease occurred earlier, at lower infused adrenaline concentrations in the CAN group compared with NAF and SAN groups. AUC for the symmetry ratio was different across the groups and was lowest in the CAN group (*p* = 0.04). Plasma adrenaline rose and potassium fell comparably in all groups.

**Conclusions/interpretation:**

Participants with CAN showed abnormal repolarisation in some measures at lower adrenaline concentrations. This may be due to denervation adrenergic hypersensitivity. Such individuals may be at greater risk of cardiac arrhythmias in response to physiological sympathoadrenal challenges such as stress or hypoglycaemia.

**Electronic supplementary material:**

The online version of this article (10.1007/s00125-020-05106-7) contains peer-reviewed but unedited supplementary material, which is available to authorised users.

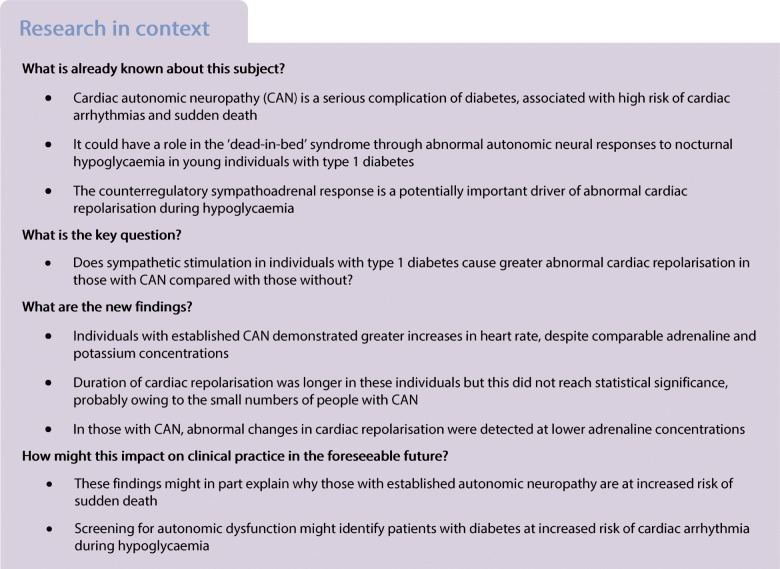



## Introduction

Cardiac autonomic neuropathy (CAN) is a serious complication of diabetes associated with increased rates of cardiac arrhythmias and sudden death [1]. Individuals with CAN exhibit intracardiac sympathetic imbalance due to cardiac parasympathetic denervation and initial sympathetic hypersensitivity leading to progressive sympathetic denervation [2]. These mechanisms might combine with the effects of hypoglycaemia to increase pro-arrhythmic risk and could identify CAN as a potential risk factor for sudden unexpected nocturnal deaths in type 1 diabetes, also known as the ‘dead-in-bed’ syndrome [3]. We previously tested this hypothesis in a study involving experimental hypoglycaemia in participants with type 1 diabetes [4]. Paradoxically, individuals with CAN showed smaller increase in QT interval duration compared to those with normal autonomic function. However, they also had attenuated sympathoadrenal responses.

In the current study, we aimed to investigate whether type 1 diabetic individuals with established autonomic neuropathy or subclinical autonomic neuropathy (SAN) would develop greater changes in cardiac repolarisation compared to those with normal autonomic function, when exposed to a standard sympathetic stimulus.

## Methods

### Research design

Individuals with type 1 diabetes, 14 men and eight women, aged between 18 and 50 years were recruited. Exclusion criteria included ischaemic heart disease, peripheral vascular disease, cerebrovascular disease, pregnancy, thyrotoxicosis, epilepsy or seizure disorder, asthma, visual impairment due to retinopathy, renal impairment due to nephropathy or current treatment with salbutamol or β-blockers. All participants gave written informed consent. The study protocol was approved by the North Sheffield Research Ethics committee.

Standard autonomic function tests were performed. See electronic supplementary material (ESM) Methods: Standard autonomic function tests for details. Responses outside age-adjusted normal ranges [5] in two or more tests were classified as abnormal standard autonomic function tests. Baroreceptor sensitivity (BRS) was calculated using the sequence method [6] (see ESM Methods: Baroreceptor sensitivity). BRS values lower than the 5th centile of age- and sex-adjusted ranges [7] were classed as abnormal. Individuals were divided into three groups: group 1—normal autonomic function (NAF; normal autonomic function tests and normal BRS); group 2—SAN (normal autonomic function tests but abnormal BRS) [6]; and group 3—established CAN (abnormal autonomic function and BRS tests).

### Adrenaline infusions

Participants were admitted in the morning. Blood glucose was maintained between 4 and 15 mmol/l using a low dose intravenous infusion of insulin (Human Actrapid, Novo Nordisk Pharmaceuticals, Crawley, UK) or boluses of 20% glucose (Freeflex, Fresenius Kabi, Runcorn, UK). Arterialised venous blood samples were collected from a retrograde cannula with the hand placed in a heated chamber at 50°C. The participants then received an incremental adrenaline (epinephrine) infusion: saline (0.9% NaCl) infusion for the first hour, followed by adrenaline infusion at 0.01 μg kg^−1^ min^−1^ for the second hour, 0.03 μg kg^−1^ min^−1^ for the third hour and finally 0.06 μg kg^−1^ min^−1^ for 30 min.

Plasma adrenaline, serum potassium, BP and heart rate were measured at baseline, at 30 min intervals during the study and 30 min after completion of the infusion (see ESM Methods: Biochemical analysis).

### ECG measurements

Five minute high resolution ECGs were recorded at 30 min intervals to determine parameters of cardiac repolarisation: QT_c_, T wave amplitude and T wave area symmetry ratio. ECG was recorded from three bipolar orthogonal electrodes and parameters of cardiac repolarisation were extracted from the combined composite wave. These included the QT_c_ interval duration and parameters describing the morphology of the T wave: T wave area symmetry ratio [8] and T wave amplitude normalised to baseline values. The Hodges formula was used to correct QT for heart rate (QT_c_). See ESM Methods: ECG measurements for further details.

### Statistical analysis

Adrenaline, potassium and cardiac repolarisation responses during the adrenaline infusion were described by summary measures for comparison between groups: AUC, time of the maximum (or minimum), absolute maximum (minimum), largest change from baseline and overall standardised variability. Groups were compared using analysis of variance where the underpinning assumptions were met or Kruskal–Wallis test. Data are presented as mean (SD) or mean (95% CI). A *p* value <0.05 was classed as significant. Statistical analysis was performed using SPSS 22 (IBM, Armonk, NY, USA). For details and power analysis see ESM Methods: Statistical analysis.

## Results

### Participant characteristics

Ten participants with NAF, seven with SAN and five with CAN participated in the study. Their mean age (SD) was 37 (6), 30 (6) and 38 (5) years, respectively (*p* = 0.04), their BMI (SD) was 24 (2), 26 (3) and 29 (5) kg/m^2^, respectively (*p* = 0.05) and the male to female ratio was 8/2, 6/1 and 0/5, respectively (*p* = 0.005). Baseline adrenaline levels (SD) were different across the groups: 0.38 (0.17), 0.28 (0.16) and 0.21 (0.07) nmol/l in the NAF, SAN and CAN groups, respectively (*p* = 0.04) (ESM Table 1).

### Biochemical analysis

**Fig. 1:**
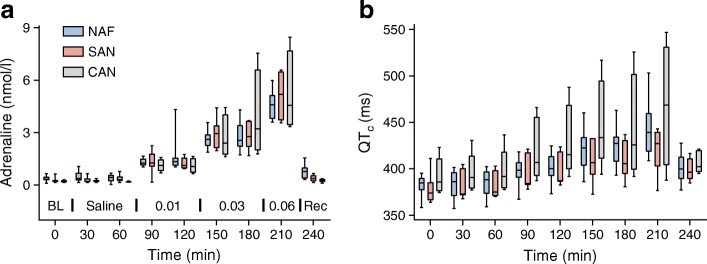
Plasma adrenaline concentration (**a**) and QT_c_ interval duration (**b**) during adrenaline infusion for participants with NAF, SAN and CAN. Data are presented using boxplots with lines at quartiles and whiskers showing the full range. The adrenaline infusion protocol is displayed in an inset (**a**, bottom), showing adrenaline infusion rate in μg kg^−1^ min^−1^. BL, baseline; Rec, recovery

Plasma adrenaline rose in all groups with no differences between the groups (Fig. 1a and ESM Table 2). Mean (95% CI) baseline adrenaline concentration for all participants was 0.34 (0.25, 0.42) nmol/l and reached a maximum 4.78 (4.08, 5.47) nmol/l at 210 min. Serum potassium fell comparably in all groups (ESM Table 3). Mean baseline potassium concentration for all participants was 4.19 (4.04, 4.35) mmol/l and decreased to 3.21 (3.10, 3.33) mmol/l at 210 min.

### Physiological variables

Baseline heart rate was 68 (95% CI 60, 76) bpm for the NAF group, 73 (59, 87) bpm for the SAN group and 84 (78, 91) bpm for the CAN group (*p* = 0.06, ESM Table 4). Heart rate increase from baseline was different across the groups: 22 (13, 32) bpm in the CAN compared with 11 (7, 15) bpm in the NAF and 10 (3, 18) bpm in the SAN groups (*p* = 0.01). Systolic and diastolic BP and their changes were comparable (ESM Table 1 and ESM Results: Physiological variables).

### Changes in ventricular repolarisation

**Fig. 2:**
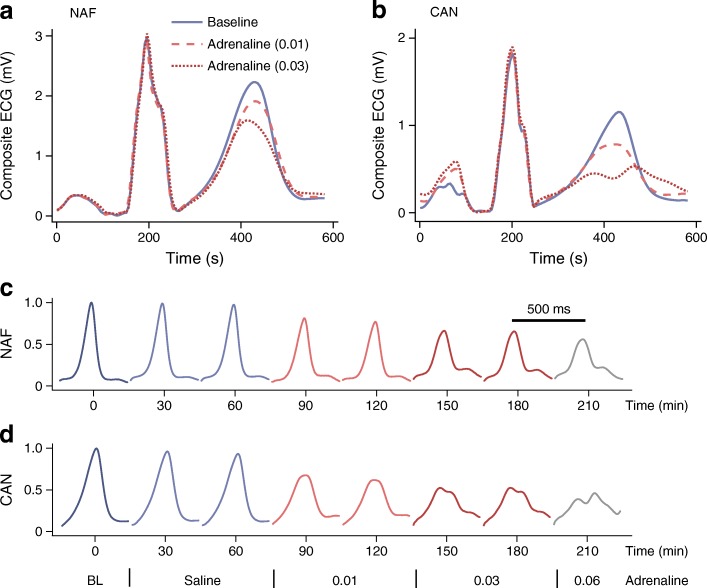
Typical composite ECG waveforms at baseline and during adrenaline infusion at 0.01 and 0.03 μg kg^−1^ min^−1^ (**a**, **b**). Changes in T wave morphology are shown for a participant with NAF (**a**) and established CAN (**b**). (**c**, **d**) ECG waveforms for the above participants are shown at all stages of the infusion protocol with different colours indicating the progressive rate of infusion. ECG T waves during adrenaline infusion demonstrate abnormal T wave morphology appearing to a greater extent and at lower dose of adrenaline in an individual with CAN (**d**) compared with NAF (**c**). The amplitude is normalised to baseline for each participant. The adrenaline infusion protocol is displayed below (**d**), showing adrenaline infusion rate in μg kg^−1^ min^−1^. BL, baseline

ECG waveforms at baseline and during adrenaline infusion are presented in Fig. 2 for one individual with NAF (Fig. 2a) and one with CAN (Fig. 2b). There was a decrease in T wave amplitude and the T waves became more symmetric with increased dose of adrenaline. In CAN the highest dose of adrenaline resulted in notched T waves (Fig. 2b, d) and the abnormal changes in morphology started at lower dose of adrenaline (Fig. 2d).

#### QT_c_ interval duration

Baseline QT_c_ was 382 (95% CI 374, 390) ms in the NAF, 378 (363, 393) ms in the SAN and 392 (367, 417) ms in the CAN groups (*p* = 0.31) (Fig. 1b and ESM Table 1). QT_c_ in all groups lengthened comparably with adrenaline infusion (ESM Table 5). The longest QT_c_ was 444 (422, 463) ms (NAF), 422 (402, 437) ms (SAN) and 470 (402, 519) ms (CAN) (*p* = 0.09).

#### Normalised T wave amplitude and T wave area symmetry ratio

Amplitude of the T wave progressively decreased across all groups to about 50% of its baseline value (ESM Table 6). Time of minimum was different across the groups: 201 (95% CI 187, 215) min (NAF), 176 (151, 201) min (SAN) and 120 (29, 211) min (CAN) (*p* = 0.02). The baseline T wave area symmetry ratio was comparable in the three groups (ESM Table 7). During the adrenaline infusion the T waves became more symmetric (decrease in symmetry ratio). Symmetry ratio equal to number 1 indicates perfect symmetry around the peak of the T wave. AUC was lowest in the CAN group (1.19 [95% CI 1.12, 1.25]) compared with the NAF (1.36 [1.19, 1.53]) and SAN (1.39 [1.22, 1.56]) groups (*p* = 0.04). Time of minimum was shortest in the CAN group (*p* = 0.01).

## Discussion

In this study, we used the infusion of adrenaline as a standardised sympathetic stimulus to investigate the effect on cardiac repolarisation among type 1 diabetic individuals with normal autonomic function, subclinical autonomic neuropathy and established autonomic neuropathy. We noted significant changes in T wave morphology in the CAN group.

QT interval correction by adjusting for heart rate can lead to an artefactual increase in QT intervals, especially at high heart rates. Thus the morphology parameters used in this study better characterise the overall repolarisation characteristics since they are less rate dependent [8]. The maximum changes in these parameters occurred at lower infused adrenaline concentrations in individuals with CAN despite comparable adrenaline levels. We also noted abnormal notched T waves in the CAN group. These changes might be explained by denervation adrenergic hypersensitivity, a phenomenon that is well described in diabetic autonomic neuropathy [9]. We found no evidence that the response to adrenaline infusion was different in the SAN group compared with the NAF group. This might be due to a functional defect in SAN in contrast to a structural one in CAN.

We did not reach target sample sizes in the SAN and CAN groups despite screening over 90 potential participants. Established autonomic neuropathy is relatively uncommon in young individuals with type 1 diabetes and patients with ischaemic heart disease and renal disease were not included for safety/ethical reasons. Thus, we cannot exclude the possibility of a type 2 error. Imbalanced sex and BMI characteristics could affect some results of our study. While QT_c_ is influenced by sex, T wave symmetry is rate- and sex-independent and less likely to be affected [8]. No corrections for multiple testing could lead to inflated false positive observations in this study. The statistical data, however, were not interpreted as definite but rather indicative of repolarisation measures that best describe and classify the changes during adrenaline infusion.

In conclusion, type 1 diabetic individuals with CAN may be more vulnerable to cardiac arrhythmias when exposed to sympathoadrenal challenges. Screening for autonomic dysfunction using bedside tests could identify patients with diabetes at increased risk of cardiac arrhythmia during hypoglycaemia. Further studies with larger numbers and better balanced groups are required to confirm our findings. Those affected might theoretically benefit from β-blockers although such a treatment might also have the potential to increase the risk of hypoglycaemia in those with impaired hypoglycaemia awareness.

## Electronic supplementary material

ESM (PDF 169 kb)

## Data Availability

The datasets generated during and/or analysed during the current study are available from the corresponding author on reasonable request.

## Abbreviations

BRS: Baroreceptor sensitivity
CAN: Cardiac autonomic neuropathy
NAF: Normal autonomic function
SAN: Subclinical autonomic neuropathy
