# Supplementary material for: Influence of cardiac autonomic neuropathy on cardiac repolarisation during incremental adrenaline infusion in type 1 diabetes
Source: Diabetologia. 2020 Feb 7;63(5):1066–71. doi: 10.1007/s00125-020-05106-7 (PMC7145773; doi:10.1007/s00125-020-05106-7)
Supplement: Supplementary file 1 — (PDF 169 kb) [file 125_2020_5106_MOESM1_ESM.pdf]

Electronic Supplementary Material

ESM Methods

Research design

Standard autonomic function tests Standard autonomic function tests (AFTs) as described by Ewing [1], using O'Brien's age adjusted normal heart rate variability ranges [2] were used. Five individual components were measured: heart rate variability at rest, heart rate response to deep breathing, to standing, and to the Valsalva manoeuvre and blood pressure response to standing.

Baroreceptor sensitivity Blood pressure (Portapres, TNO biomedical instrumentation, Amsterdam, Netherlands) and ECG were recorded simultaneously for 5 minutes. Data were digitised (DI-158U, DATAQ instruments, USA) with a sampling frequency of 1000Hz. Patients rested supine for 15 minutes prior to measurement. Spontaneous BRS was calculated using the sequence method (Nevrokard, v5.1.3, Intellectual Services, Slovenia) [3]. Total baroreceptor sensitivity ($ms.mmHg^{-1}$) was calculated by averaging the regression coefficients of the "up" and "down" sequences.

Biochemical analyses Blood was collected into tubes containing a clot accelerator and separation gel (BD Vacutainer®SST™ II Advance). The samples were centrifuged at 3000g at room temperature for 10 min. Assays on the resulting serum were performed at 37°C using an automated system (SYNCHRON LX® System). Samples for adrenaline (6 ml) were taken into chilled lithium heparin tubes containing 50 microlitres of EGTA/glutathione preservative and centrifuged at 4°C, 3000rpm for 10 minutes. The resulting supernatant was stored at -80°C until analysed by high performance liquid chromatography (HPLC) [4].

ECG measurements

High resolution ECG measurements were recorded from three bipolar orthogonal electrodes (X, Y and Z) at 12-bit resolution and 750 samples per second using a custom built high resolution system. Beat averaging was performed over a period of 5 minutes using a template matching scheme [5]. The averaged beats were upsampled to 1000Hz, filtered using a 40Hz low-pass filter and the composite wave was calculated as the square root of $X^2+Y^2+Z^2$. Semi-automatic custom-built software was used to calculate the QT interval based on the tangent method and to extract the parameters describing the morphology of the T wave. Onset and offset of the T wave were defined at the intersection of the isoelectric line and the tangents to both sides of the T wave [6]. The T wave area symmetry ratio (T_{sym}) was calculated as the ratio

of the area under the T wave before and after the peak [7]. The T wave amplitude was measured from the isoelectric line and was normalised to the amplitude registered at the baseline recording. All markers were manually checked and adjusted if necessary by an observer blinded to the participant characteristics.

The Hodges formula for rate correction has been shown to be less heart rate dependent than both the Bazett and Fridericia formulas in patients with normal and prolonged cardiac repolarisation [8] and has been specifically suggested to investigate changes in QT_c in drug induced long QT syndrome [9].

Statistical analysis

Analysis of variance was used to investigate the differences in outcome measures between the NAF, SAN and CAN groups. Alternatively, the Kruskall-Wallis test was used where the assumptions underpinning analysis of variance were not met. These included: testing the normality of residuals (Shapiro-Wilk test and inspection of QQ-plots) and homogeneity of variance (Levene's test). The Fisher's exact test was used to compare the numbers of male and female participants between the groups.

A prospective power calculation for the primary output QT_c was performed based on a previous adrenaline infusion study in healthy individuals [10]. At least 10 subject per group were deemed necessary to provide 80% power to detect a clinically relevant difference of 36 ms in maximum QT_c at significance level 0.05. Retrospective power calculations revealed that the power to detect a 36 ms difference in QT_c dropped to 49% when the actually recruited numbers of subjects were used (10, 7 and 5). In contrast, the retrospective powers using the actual subject numbers for summary measures time to minimum in normalised T wave amplitude and time to minimum in T wave area symmetry ratio were 78% and 82%, respectively. Retrospective power was calculated by simulations, where parameter estimates based on the study data were used in an unbalanced one-way ANOVA setup with 3 groups. Power calculations were performed using R 3.6.1.

ESM Results

Physiological variables

Blood pressure Baseline systolic and diastolic pressures are given in ESM Table 1. Systolic blood pressure increased during the adrenaline infusion in all groups with a mean maximum change (95% CI): 12.3 (7.6, 17.0), 13.3 (8.3, 18.3) and 14.8 (1.6, 28.0) mmHg in the normal autonomic function, subclinical autonomic neuropathy and established CAN groups

respectively. There was no significant difference between the groups (p=0.83). Diastolic blood pressure fell slightly in all groups during the infusion with mean (95% CI) falls of: -7.7 (-10.4, -5.0), -7.6 (-11.7, -3.4) and -9.2 (-17.7, -0.7) mmHg in the NAF, SAN and CAN groups respectively, with no significant differences between the groups (p=0.82).

ESM References

1. Ewing DJ, Clarke BF (1982) Diagnosis and management of diabetic autonomic neuropathy. Br Med J (Clin Res Ed) 285(6346):916–8
2. O'Brien IAD, O'Hare P, Corrall RJM (1986) Heart rate variability in healthy subjects: effect of age and the derivation of normal ranges for tests of autonomic function. Br Hear J 55(4):348–54. https://doi.org/10.1136/hrt.57.1.109-a
3. Frattola A, Parati G, Gamba P, et al (1997) Time and frequency domain estimates of spontaneous baroreflex sensitivity provide early detection of autonomic dysfunction in diabetes mellitus. Diabetologia 40(12):1470–1475. https://doi.org/10.1007/s001250050851
4. Forster CD, Macdonald IA (1999) The assay of the catecholamine content of small volumes of human plasma. Biomed Chromatogr 13(3):209–215. https://doi.org/10.1002/(SICI)1099-0801(199905)13:3<209::AID-BMC820>3.0.CO;2-Z
5. Alperin N, Sadeh D (1986) An improved method for on-line averaging and detecting of ECG waveforms. Comput Biomed Res 19(3):193–202. https://doi.org/10.1016/0010-4809(86)90015-7
6. Postema PG, Wilde AAM (2014) The measurement of the QT interval. Curr Cardiol Rev 10(3):287–94. https://doi.org/10.2174/1573403X10666140514103612
7. Merri M, Benhorin J, Alberti M, Locati E, Moss AJ (1989) Electrocardiographic quantitation of ventricular repolarization. Circulation 80(5):1301–8. https://doi.org/10.1161/01.CIR.80.5.1301
8. Chiladakis J, Kalogeropoulos A, Arvanitis P, Koutsogiannis N, Zagli F, Alexopoulos D (2010) Heart rate-dependence of QTc intervals assessed by different correction methods in patients with normal or prolonged repolarization. Pacing Clin Electrophysiol 33(5):553–560. https://doi.org/10.1111/j.1540-8159.2009.02657.x
9. Chiladakis J, Kalogeropoulos A, Arvanitis P, Koutsogiannis N, Zagli F, Alexopoulos D (2010) Preferred QT correction formula for the assessment of drug-induced QT interval prolongation. J Cardiovasc Electrophysiol 21(8):905–913. https://doi.org/10.1111/j.1540-8167.2010.01738.x
10. Lee S, Harris ND, Robinson RT, Yeoh L, Macdonald IA, Heller SR (2003) Effects of adrenaline and potassium on QTc interval and QT dispersion in man. Eur J Clin Invest 33(2):93–98. https://doi.org/10.1046/j.1365-2362.2003.01123.x

ESM Table 1: Baseline characteristics of participants.

	Normal autonomic function NAF (*n*=10)	Subclinical CAN SAN (*n*=7)	Established CAN CAN (*n*=5)	*p* value
Age (years)	36.6 (5.6)	30.1 (6.0)	38.0 (5.0)	0.04
Male/Female	8M/2F	6M/1F	0M/5F	0.005[a]
BMI (kg/m^2)	23.7 (1.9)	26.5 (3.1)	28.7 (5.2)	0.05[b]
HbA_{1c} (%) [c]	8.9 (1.4)	8.0 (0.9)	9.9 (1.9)	0.12
(mmol/mol) [c]	73.7 (15.6)	64.3 (10.0)	84.9 (20.3)	0.12
Creatinine (µmol/l)	71 (9)	71 (14)	66 (8)	0.64
Duration of diabetes (years)	14.8 (11.9)	15.7 (9.5)	18.0 (5.7)	0.85
Heart rate (bpm)	67.9 (11.3)	72.7 (15.4)	84.4 (5.2)	0.06
RR interval (ms)	909 (171)	855 (164)	713 (43)	0.09
QT (ms)	368 (25)	356 (37)	349 (26)	0.46
QT_c Hodges (ms)	382 (11)	378 (16)	392 (20)	0.31
Systolic BP (mmHg)	120 (11)	123 (14)	117 (14)	0.72
Diastolic BP (mmHg)	74 (8)	71 (7)	72 (8)	0.81
Plasma adrenaline (nmol/l) [d]	0.38 (0.17)	0.28 (0.16)	0.21 (0.07)	0.04[b]

Data are mean (SD). [a] Fisher's exact test, [b] Kruskall-Wallis test, [c] $n = 6$ for SAN group, [d] n=4 for CAN group.

ESM Table 2: Plasma adrenaline summary measures during adrenaline infusion

Adrenaline (nmol/l)		Estimated mean value	95% CI	*p* value for difference between groups
Baseline	NAF	0.38	0.25 to 0.50	0.04[a]
	SAN	0.28	0.13 to 0.43	
	CAN	0.21	0.14 to 0.28	
AUC	NAF	1.17	1.51 to 2.03	0.87
	SAN	1.83	1.39 to 2.27	
	CAN	1.69	0.99 to 2.39	
Maximum value	NAF	4.63	4.07 to 5.19	0.70[a]
	SAN	5.15	3.88 to 6.43	
	CAN	5.76	3.04 to 8.47	
Time of maximum (min)	NAF	201	181 to 221	0.17[a]
	SAN	210	210 to 210	
	CAN	192	159 to 225	
Biggest change from baseline	NAF	4.25	3.71 to 4.80	0.60[a]
	SAN	4.87	3.60 to 6.15	
	CAN	5.54	2.88 to 8.20	
Variability	NAF	0.33	0.18 to 0.49	0.46[a]
	SAN	0.41	0.15 to 0.68	
	CAN	0.46	0.22 to 0.71	

n=10 (NAF), *n*=7 (SAN) and *n*=4 (CAN). [a] Kruskall-Wallis test.

ESM Table 3: Plasma potassium summary measures during adrenaline infusion

Potassium (mmol/l)		Estimated mean value	95% CI	*p* value for difference between groups
Baseline	NAF	4.16	3.98 to 4.34	0.68
	SAN	4.20	4.04 to 4.36	
	CAN	4.28	3.89 to 4.67	
AUC	NAF	3.81	3.66 to 3.96	0.83
	SAN	3.87	3.70 to 4.05	
	CAN	3.84	3.58 to 4.10	
Minimum value	NAF	3.32	3.15 to 3.49	0.38[a]
	SAN	3.37	3.13 to 3.61	
	CAN	3.18	2.90 to 3.46	
Time of minimum (min)	NAF	201	181 to 221	0.91[a]
	SAN	201	180 to 222	
	CAN	198	165 to 231	
Biggest change from baseline	NAF	-0.84	-1.00 to -0.68	0.56[a]
	SAN	-0.83	-1.03 to -0.62	
	CAN	-1.10	-1.68 to -0.52	
Variability	NAF	0.19	0.14 to 0.25	0.13
	SAN	0.21	0.15 to 0.28	
	CAN	0.13	0.05 to 0.21	

n=10 (NAF), *n*=7 (SAN) and *n*=4 (CAN). [a] Kruskall-Wallis test.

ESM Table 4: Heart rate summary measures during adrenaline infusion

Heart rate (bpm)		Estimated mean value	95% CI	*p* value for difference between groups
Baseline	NAF	68	60 to 76	0.06
	SAN	73	59 to 87	
	CAN	84	78 to 91	
AUC	NAF	71	63 to 78	0.003
	SAN	75	64 to 87	
	CAN	93	86 to 101	
Maximum value	NAF	79	72 to 86	0.001
	SAN	83	72 to 94	
	CAN	107	94 to 119	
Time of maximum (min)	NAF	201	187 to 215	0.99[a]
	SAN	193	151 to 235	
	CAN	198	165 to 231	
Biggest change from baseline	NAF	11	7 to 15	0.01
	SAN	10	3 to 18	
	CAN	22	13 to 32	
Variability	NAF	8	3 to 13	0.16[a]
	SAN	10	4 to 17	
	CAN	4	1 to 7	

n=10 (NAF), *n*=7 (SAN) and *n*=4 (CAN). [a] Kruskall-Wallis test.

ESM Table 5: QT_c interval summary measures during adrenaline infusion

QT_c (ms)		Estimated mean value	95% CI	*p* value for difference between groups
Baseline	NAF	382	374 to 390	0.31
	SAN	378	363 to 393	
	CAN	392	367 to 417	
AUC	NAF	405	394 to 416	0.31[a]
	SAN	396	381 to 410	
	CAN	423	380 to 455	
Maximum value	NAF	444	422 to 463	0.09
	SAN	422	402 to 437	
	CAN	470	402 to 519	
Time of maximum (min)	NAF	204	190 to 216	0.41[a]
	SAN	189	154 to 226	
	CAN	186	145 to 233	
Biggest change from baseline	NAF	62	42 to 81	0.15
	SAN	44	27 to 61	
	CAN	78	26 to 130	
Variability	NAF	13.1	7.3 to 18.9	0.18[a]
	SAN	14.5	8.4 to 20.5	
	CAN	25.6	4.8 to 46.4	

n=10 (NAF), *n*=7 (SAN) and *n*=4 (CAN). [a] Kruskall-Wallis test.

ESM Table 6: Normalised T wave amplitude summary measures during adrenaline infusion

Normalised T wave amplitude (T_{amp})		Estimated mean value	95% CI	*p* value for difference between groups
Baseline	NAF	1.00	-	
	SAN	1.00	-	-
	CAN	1.00	-	
AUC	NAF	0.78	0.73 to 0.83	
	SAN	0.81	0.74 to 0.87	0.87[a]
	CAN	0.82	0.63 to 1.00	
Minimum value	NAF	0.46	0.37 to 0.55	
	SAN	0.61	0.48 to 0.74	0.16
	CAN	0.50	0.23 to 0.77	
Time of minimum (min)	NAF	201	187 to 215	
	SAN	176	151 to 201	0.02[a]
	CAN	120	29 to 211	
Biggest change from baseline	NAF	-0.54	-0.63 to -0.45	
	SAN	-0.39	-0.52 to -0.26	0.16
	CAN	-0.50	-0.77 to -0.23	
Variability	NAF	0.07	0.05 to 0.09	
	SAN	0.07	0.05 to 0.10	0.02[a]
	CAN	0.17	0.10 to 0.25	

n=10 (NAF), *n*=7 (SAN) and *n*=4 (CAN). [a] Kruskall-Wallis test.

ESM Table 7: T wave area symmetry ratio summary measures during adrenaline infusion

T wave area symmetry ratio (T_{sym})		Estimated mean value	95% CI	*p* value for difference between groups
Baseline	NAF	1.48	1.31 to 1.65	
	SAN	1.62	1.41 to 1.83	0.28
	CAN	1.65	1.46 to 1.85	
AUC	NAF	1.36	1.19 to 1.53	
	SAN	1.39	1.22 to 1.56	0.04[a]
	CAN	1.19	1.12 to 1.25	
Minimum value	NAF	0.88	0.68 to 1.09	
	SAN	0.92	0.73 to 1.11	0.15
	CAN	0.65	0.43 to 0.87	
Time of minimum (min)	NAF	204	195 to 213	
	SAN	206	195 to 216	0.01[a]
	CAN	156	101 to 211	
Biggest change from baseline	NAF	-0.65	-0.92 to -0.37	
	SAN	-0.71	-1.04 to -0.38	0.17
	CAN	-1.05	-1.53 to -0.56	
Variability	NAF	0.22	0.11 to 0.33	
	SAN	0.21	0.14 to 0.29	0.55[a]
	CAN	0.22	0.17 to 0.28	

n=10 (NAF), *n*=7 (SAN) and *n*=4 (CAN). [a] Kruskall-Wallis test.
